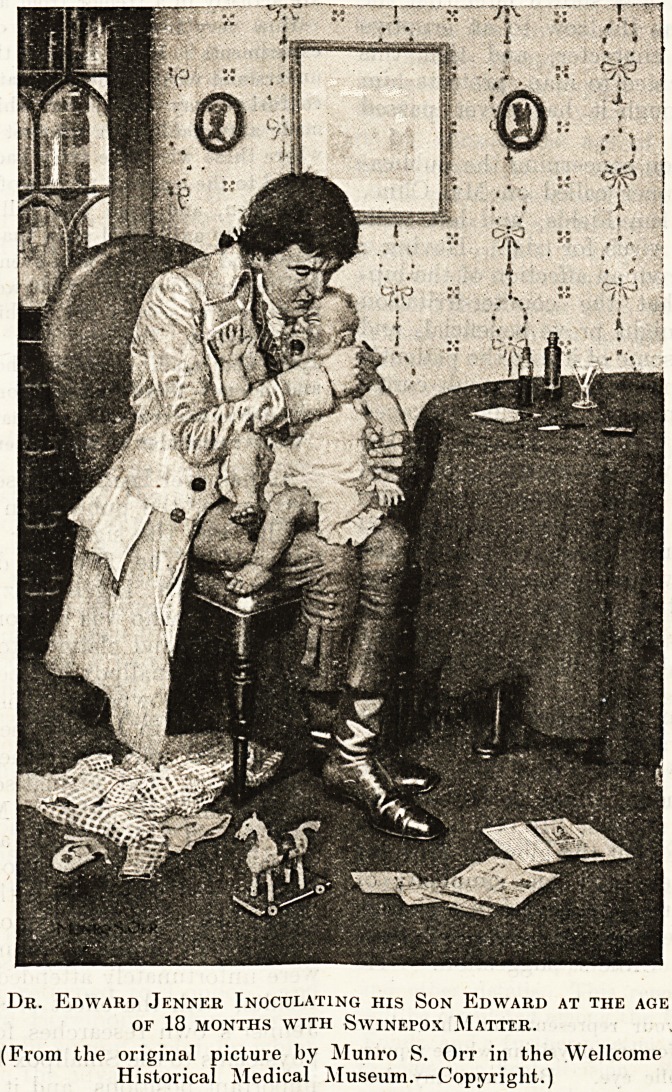# Edward Jenner—I

**Published:** 1921-11

**Authors:** C. J. S. Thompson

**Affiliations:** M.B.E. Curator of the Wellcome Historical Medical Museum


### EDWARD JENNER.—I.

### THE DISCOVERER OF VACCINATION.

By C. J. S. Thompson, M.B.E., Curator of the Wellcome Historical Medical Museum.

The close of the eighteenth century saw the dawn of a new era in preventive medicine, and one that will ever be memorable for the discovery of vaccination by Dr. Edward Jenner. His name still lives as the vanquisher of smallpox, which for centuries before his time had ravaged the world.

The principle of inoculation for the prevention of disease dates back to a period of great antiquity, and as a protection against smallpox it appears to have had its origin in India, where inoculation with actual virus taken from the smallpox pustule is said to have been known over 1,000 years before the time of Christ. The practice passed from India to China, where as early as the fourteenth century there is record of a kind of inoculation having been used for smallpox. Savage races in various parts of Africa appear also to have known and practised inoculation as a protection from the disease. From Asia and Africa the knowledge of inoculation passed into Europe by way of Greece and the coast of the Bosphorus to Constantinople, where it was well known at the latter part of the seventeenth century. In the early eighteenth century it reached Western Europe, and in 1721 an arm-to-arm variolation was practised in England. In 1746 a hospital was established in London for systematically carrying out inoculation, but the risks attending it prejudiced the majority of people against the practice. The terrible mortality resulting from frequent epidemics led scientific workers of the period to try and discover some safer method of preventing the disease.

Edward Jenner at the age of 35.
(Wellcome Historical Medical Museum.—Copyright.)

Edward Jenner, who was destined to solve the problem, was born on May 17,1749, at the Vicarage of Berkeley in Gloucestershire, and was the third son of the Rev. Stephen Jenner, M.A., Vicar of that place. Just at this time inoculation was being vigorously advocated as a preventive of smallpox, and was commonly practised in England. When he was but eight years of age Edward Jenner's parents decided that he should be inoculated, and he tells us that for six weeks he was bled and purged, kept on a low diet, was dosed with medicine, and then removed to one of the so-called "inoculation stables" and haltered up with others in a terrible state of disease. He was fortunate to escape with a mild attack, but it affected his health for many years afterwards, and it is probable that the experience he then went through made such an impression upon his mind that he eventually began his investigations on some less dangerous method of preventing the disease.

At the age of thirteen he decided to follow the profession of medicine, and was apprenticed by his father to Messrs. Ludlow, a firm of surgeons in Sodbury, near Bristol, with whom he remained for six years. It was during this period of apprenticeship that one day a young countrywoman came to seek medical advice, and, the subject of smallpox having been mentioned, she said, "I cannot take it, for I have had cowpox." This statement seems to have made a deep impression on Edward Jenner, and doubtless set him thinking why this should be. It marked out in his mind a new line of research for future exploration, and the seed so planted bore fruit of the greatest importance to mankind.

On attaining the age of twenty-one, he came to London and entered as a house-pupil with the famous surgeon, John Hunter, in whose family he resided for two years, and became his favourite assistant. Hunter's firm and independent character produced a deep and lasting impression on Jenner, who followed in his footsteps in his thirst for knowledge, honesty of purpose, and powers of versatility. He assisted in forming the great anatomical museum which now forms part of the Royal College of Surgeons in England. It is said he often discussed the subject of smallpox with the great anatomist, and on one occasion, when relating his hopes and fears of the possibility of substituting vaccination for inoculation, Hunter's characteristic reply was: "Don't *think*, Jenner, but *try*." On leaving London, he settled down to practise in Berkeley, his native village, and two

**Edward Jenner—(*cont.*).**

years later found time to take up the study of cowpox, which, owing to his frequent intercourse with farmers and those engaged in the fields in this pastoral district, was no doubt constantly in his mind.

We can picture him at the age of thirty-one as a man under middle size, but robust, active, and of good physique. He was particular in his dress, and usually wore a blue coat with yellow buttons, buckskins, well-polished jockey boots with handsome silver spurs, and carried a smart whip with a silver handle. His hair, after the fashion of the time, was done up in a club, and he wore a broad-brimmed hat. His most intimate friend was a Mr. Gardner, a man of sympathetic character, of whom Jenner made a confidant, often discussing his work of research. He tells us how one day when riding together he first imparted to his friend the result of his study into the natural history of cowpox. Jenner believed at that time the disease took its origin from the heel of the horse, and he specified the different kinds of disease which attacked milkmen when they handled infected cows. He dwelt upon that variety which appeared to afford protection against smallpox, and with deep and anxious emotion mentioned his hope of being able to propagate that variety from one human being to another, until he had disseminated the practice all over the world to the total extinction of that terrible disease. We can imagine him drawing his horse closer to his friend's and bending over with impressive words saying, "Gardner, I have entrusted a most important matter to you which I firmly believe will prove of essential benefit to the human race. I know you, and should not wish what I have stated to be brought into conversation, for should anything untoward turn up in my experiments I should be made—particularly by my medical brethren—the subject of ridicule, for I am the mark they all shoot at."

It was at this time Jenner concluded his researches into what is commonly called in England "The Grease," a disease which attacks horses' heels and is well known to farriers, and identified it as the same as cowpox and smallpox. This happened one day when, accompanied by his nephew, George Jenner, he was examining a horse with diseased heels, and, pointing to the infected part, he cried, "There is the source of smallpox. I have much to say on that subject, which I hope in due time to give to the world." He satisfied himself that the two forms of disease had been hitherto confounded, and that only one gave protection against smallpox. It was not until 1780, however, that he was enabled, after much study and research, to unravel many of the perplexing obscurities in connection with the truth of the traditions respecting cowpox.

Jenner's next step was to ascertain that the true cowpox itself only protects when communicated at a particular stage of the disease. Just at this time, however, there was little opportunity of studying cowpox in that part of the country, as few cases had occurred in Gloucestershire. He had, therefore, no opportunity of inoculating the disease and so putting his theories to the test; but he steadily pursued his investigations, and in 1788 he had a drawing made of the hand of a milkmaid with cowpox, which he took with him to London to Sir Everard Home, who agreed that it was both interesting and curious, and the subject began to be talked about in medical circles in London.

On March 6, 1788, Jenner married Miss Kingscote, the daughter of Mr. Anthony Kingscote, with whom he had been long acquainted. Their first child, Edward, was born in January 1789.

While investigating the subject of vaccine inoculation, he made some experiments with swinepox, which he believed to be of similar origin to common variolæ. In order to prove this, in 1790, Jenner took an important and heroic step. Finding no other subject, he resolved to inoculate his own son, Edward (who was then a baby about eighteen months old), with some swinepox matter which he had collected. We can imagine that Jenner watched the result with the greatest interest, and noticed that the progress of the disease seemed similar to that arising from the insertion of true smallpox matter when the attack was slight. No harm apparently resulting to the child, on April 7, 1791, he again inoculated him, and, although a vesicle appeared and there was some erysipelas, it quickly faded away, and the child showed no sign of indisposition the whole time.

Thus for the time Jenner's researches were at a standstill. In 1796, however, the opportunity occurred for a most important experiment. He was informed that cowpox had broken out in a farm near Berkeley and a dairymaid named Sarah Nelmes had contracted the disease. Jenner at once seized the opportunity, and resolved to put his theories to a practical test. On May 14, 1796, he took some matter from a pustule on the girl's hand and inserted it by means of superficial incisions into the arm of a healthy boy about eight years of age named James Phipps. The experiment was successful, and even surpassed Jenner's anticipations, the result being described as similar to that produced by inoculation with variolous matter. The whole died away, leaving scabs and subsequent eschars. After a period of six weeks had elapsed Jenner determined to put his theory to the test by inoculating the boy with smallpox matter, and on July 1 of the same year, by means of punctures and slight incisions, he inoculated him with variolous lymph, and was delighted to see that no smallpox followed. This culminating point in Jenner's researches was the result of more than thirty years' reflection and study.

This historic result he communicated to his friend Gardner, and may be told in his own words:—

"As I promised," he writes, "to let you know how I proceeded in my inquiry into the nature of that singular disease the cowpox, and, being fully satisfied how much you feel interested in its success, you will be gratified in hearing that I have at length accomplished what I have been so long waiting for, the passing of the Vaccine Virus from one human being to another by the ordinary

**Edward Jenner—(*cont.*).**

mode of inoculation. A boy by the name of Phipps was inoculated in the arm from a pustule on the hand of a young woman who was infected by her master's cows. Having never seen the disease but in its casual way before, that is, when communicated from the cow to the hand of the milker, I was astonished at the close resemblance of the pustules. But now listen to the most delightful part of my story. The boy has since been inoculated for the smallpox, which, as I ventured to predict, produced no effect. I shall now pursue my experiments with redoubled ardour. Believe me, Yours very sincerely, Edward Jenner. Berkeley, July 19, 1796."

With characteristic caution and accuracy Jenner decided to confirm his experiments and make his discovery certain before putting the facts to the world, and so he resolved to repeat it, but unfortunately the disappearance of cowpox in the dairies again caused a delay. In the meanwhile, he resolved to prepare a paper on the subject to send to the Royal Society in London.

Early in the year 1797, owing to another outbreak of cowpox in the district, Jenner's opportunity again occurred. He confirmed his previous experiments, and inoculated three other persons with success. He then completed his paper and revised it for publication, sending the manuscript to the Royal Society. It was submitted to the Council, which, on consideration, after some time returned it to him, as they thought the evidence was not strong enough to warrant publication in their "Transactions." Jenner, with unshaken faith and in the firm conviction that his results were conclusive, resolved to publish the paper himself, and thus make his discovery known to the world. Before doing this he journeyed to London with his wife and daughter on April 24, 1798, for the purpose of exhibiting cowpox and demonstrating to his professional colleagues the accuracy of his researches and the truth of his assertions. He remained in London until July 14, and left on that day, bitterly disappointed, as he had been unable during the three months' visit to find a single person who would submit to vaccination.

Dr. Edward Jenner Inoculating his Son Edward at the age of 18 months with Swinepox Matter.

(From the original picture by Munro S. Orr in the Wellcome Historical Medical Museum.—Copyright.)

About the end of June 1798 his manuscript was printed, with additions, in the form of a pamphlet entitled "Inquiry into the Cause and Effects of the Variolæ Vaccinæ, a Disease discovered in some of the Western Counties of England, particularly Gloucestershire, and known by the name of the Cowpox." In this historic treatise, which led to such important results, Jenner began by referring to the disease of the horse called by farriers the "grease," which he describes as

"An inflammation and swelling in the heel, from which issues matter possessing properties of a very peculiar kind. It is capable of generating a disease in the human body (after it has undergone the modification which I shall presently speak of) which bears so strong a resemblance to the smallpox, that I think it highly probable it may be the source of that disease. In this dairy country a great number of cows are kept. The office of milking is here performed indiscriminately by both men and maid servants. One of the former having perhaps been appointed to apply dressings to the heels of a horse affected with the 'Grease,' and not paying due attention to cleanliness, incautiously bears his part in milking the cows with some particles of the infectious matter adhering to his fingers. Should this be the case, it commonly happens that a disease is communicated to the cows, and from the cows to the dairymaids, which pretty rapidly spreads until most of the cattle and domestics of the farm feel its unpleasant consequences."

Jenner thus accounts for the origin of cowpox, the characters of which he then describes in detail. He believed that virus from the horse's heels was intensified by being passed through the cow, on the ground that the horse so rarely affects his

**Edward Jenner—(*cont.*).**

dresser with sores, while the milkman rarely escapes infection from the cow. Among the cases which he describes is that of his second son, Robert FitzHarding Jenner, an infant of eleven months, and of several other children, who were vaccinated on April 12, 1798, with matter from the arm of Hannah Exell. It is particularly specified that Robert Jenner did not receive the infection. He concludes his remarks with the assertion that the cowpox protects the human constitution from the infection of smallpox is proved by the facts adduced. That the disease commonly called the "grease" was the source of cowpox was subsequently corrected by Jenner. It was shown later that the horse is liable as well as the cow to an eruptive disease of a variolous character, and that this disease, when communicated to man, protects him from smallpox even though it has never passed through the cow.

While he was in London concerning the publication of his pamphlet Jenner called on Mr. Cline, a surgeon in Lincoln's Inn Fields, and left with him some of the cowpox virus for trial. Having a young patient suffering from an affection of the hip-joint, Cline thought that the counter-irritation excited by the cowpox might prove beneficial, and in July 1798 he inserted some of it into the patient's hip by means of two punctures. The result corroborated Jenner's experiments; the child sickened on the seventh day, and the fever subsided on the eleventh. The patient was afterwards inoculated with smallpox matter in three places without contracting the disease, and Cline, writing on August 2, 1789, to Jenner, states: "I think the substitution of the cowpox poison for smallpox promises to be one of the greatest improvements that has ever been made in medicine. The more I think on the subject the more I am impressed with its importance."

Cline, convinced from the success of his first trial of the inestimable value of Jenner's discovery, advised him to leave the country and take a house in the West End of London, where he felt sure he would reap a reward of at least £10,000 a year as the result of his practice; but the glowing prospect did not appeal to Jenner, and his simplicity of mind and unselfish nature is evidenced in a charming letter which he wrote from Cheltenham on September 29 concerning Cline's suggestion. He says:—

It is very clear from your representation that there is now an opening in town for any physician whose reputation stood fair in the public eye. But here, my dear friend, is the rub. Shall I, who even in the morning of my days sought the lowly and sequestered paths of life, the valley and not the mountain, shall I, now my evening is fast approaching, hold myself up as an object for fortune and for fame? Admitting it as a certainty that I obtain both, what stock should I add to my little fund of happiness? My fortune, with what flows in from my profession, is sufficient to gratify my wishes; indeed, so limited is my ambition and that of my nearest connections, that were I precluded from future practice I should be enabled to obtain all I want. And as for fame, what is it? a gilded butt, for ever pierced with the arrows of malignancy. The name of John Hunter stamps this observation with the signature of truth. However, this I promise you, that as soon as my engagements here cease, you shall see me in town. In my last letter I told you how much I was perplexed; my perplexity really amounts to agitation. On the one hand unwilling to come to town myself for the sake of practice, and on the other, fearful that the practice I have recommended may fall into the hands of those who are incapable of conducting it, I am thrown into a state that was at first not perceptible as likely to happen to me; for, believe me, I am not callous to all the feelings of those wounds which, from misrepresentation, might fall on my reputation; on the contrary, no nerves could feel more acutely; and they now are actually in a tremor from anticipation.

How very few are capable of conducting physiological experiments! I am fearful that before we thoroughly understand what is cowpox matter, and what is not, some confusion may arise; for which I shall, unjustly, be made answerable. In the first place, instances will occur where those who have truly had the disease shall be subjected to the common process of inoculation, inflammation, vesication, and even pus will appear on the wounded part. The axilla will show that the lymphatics have been active and the system may even, in a very limited degree, feel the consequence. What would the enemies to the improvement of science say to this? I leave you to answer this question. But the very same thing has happened again and again to those who have had the smallpox; and do not those (nurses, for example) who are much exposed to the contagion of smallpox . . .

(The remainder of this letter is, unfortunately, lost.)

As has ever been the case at the advent of great discoveries, the publication of Jenner's successful results was the signal for an outburst of adverse criticism. The first to denounce Jenner's discovery was Dr. Ingenhousz, a well-known scientist of the time, who was a strong opponent of the cowpox theory, and claimed to bring certain cases to light where smallpox had been contracted after inoculation by cowpox. The leading scientific and medical men in London next took up the subject, and several questioned the accuracy of Jenner's observations, and stigmatised his doctrines as conjectural and ridiculous. Meanwhile, Ingenhousz, who proved a formidable antagonist, did much to weaken Jenner's position. Others such as Pearson and Woodville, although adopting Jenner's ideas, endeavoured to exploit them on lines of their own, which proved a failure. Their experiments were unfortunately attended with somewhat serious results, with the effect of stopping the progress of Jenner's own researches for a time. Both, being physicians to the Smallpox Hospital in London, held important positions, and it is said that the experiments they commenced to carry out on vaccination were so carelessly performed that they were practically useless. It was further said that the vaccine they used was actually disseminating the disease they wished to prevent.

On hearing this, Jenner, fearing that their failures would seriously rebound upon him, decided to leave his country home and come to London. In the early part of the year 1799 he came to the Metropolis, and at once set to work to rescue his

**Edward Jenner—(*cont.*).**

discovery from destruction and to expose the errors which had been committed by his imitators. He soon gathered round him a numerous band of enthusiastic supporters, and they at once set to work to try and counteract the evil done to their cause. Writing to his friend Gardner on March 7, 1799, he reveals the condition and agitation of his mind at this period. From this letter I extract the following:—

There never was a period in my existence when my situation called so loudly for the assistance of my literary friends as the present. Though my barque will, with flying colours, reach the shore at last, yet it is now in a storm.

I am beset on all sides with snarling fellows, and so ignorant withall, that they know no more of the disease they write about than the animals which generate it. . . . Standing as I do before so awful a tribunal, my friends will volunteer their counsel and *immediately* appear in court. . . .

He first took up his residence in Norfolk Street, London, and on the 23rd had an interview with Dr. Woodville, who informed him that he had vaccinated upwards of 200 patients. He remained in London until June 14, 1799, and during his stay had many and important interviews with most of the leading medical men resident in London, and eventually went back to Gloucestershire to procure fresh cowpox virus from the country. In 1799 Woodville had tried a succession of arm-to-arm vaccinations, and found that the virus could be passed from one person to another and still yield the same result. This method of human vaccination proving successful, it became commonly adopted in practice.

Meanwhile, Pearson, who was ambitious to be in the forefront of the investigations, decided to establish an institution of his own for the inoculation of cowpox, and appointed a Vaccine Board, of which he himself was the principal, and the Duke of York consented to become the patron. He condescendingly wrote to Jenner offering to make him an "extra corresponding physician," but he, naturally resenting this, declined the offer, and returned to Berkeley in order to complete a second paper, on which he was engaged in reply to the criticisms of his opponents. This done, it was shortly after published with the title: "A Continuation of Facts and Observations Relative to the Variolæ Vaccinæ." Soon after the publication of this pamphlet he again returned to London, and communicated with Lord Egremont, asking for an interview so that "he might submit a plan by which the country might derive the advantages of his discovery and profit by his advice." At this time, he also had audience with the Duke of Clarence, and eventually submitted a scheme for the establishment of a public institution for vaccine inoculation. He ultimately succeeded in inducing the Duke of Clarence and Lord Egremont to withdraw from Pearson's projected institution, and was presented by Lord Berkeley to King George III, the Queen, and the Prince of Wales, who expressed great interest. Their encouragement gave him fresh hope, and materially aided the spread of the vaccination propaganda throughout the country.

The practice of vaccination was introduced and first taken up in America by Dr. Waterhouse, of Cambridge, Massachusetts. He made it known in an article he published in the "Columbian Sentinel" in March 1797, entitled "Something Curious in the Medical Line." Thus with characteristic energy and enterprise did the Americans grasp a discovery which had only just been announced in the land of its birth. At a meeting of the American Academy of Arts and Sciences, presided over by John Adams, then President of the United States of America, the subject was brought forward and attentively considered, and no time was lost in endeavouring to procure a supply of vaccine lymph. It was not until June 1800, however, that a supply was sent to America, and on July 8 of that year Waterhouse vaccinated one of his sons of the age of five. This boy was the first person to be vaccinated in America. The result proving successful as compared with Jenner's experience, Waterhouse vaccinated several other members of his family, and also subjected them afterwards to smallpox inoculation. The children resisted the disease even when subjected to the most crucial tests, to the delight of Waterhouse, who exclaimed: "One fact in such cases is worth a thousand arguments."

Waterhouse did a great deal to further the practice of vaccination and Jenner's discovery, and was anxious that its effects should be diffused throughout the entire continent of America. His efforts attracted the attention of Thomas Jefferson, then President of the United States, who took a considerable interest in the subject. Jefferson had some of the members of his family vaccinated in August 1801 at Washington, and from his own family he supplied Dr. Gantt with a small quantity of vaccine lymph. Thus the seed of vaccination was planted in the capital of the United States.

(*To be concluded next month.*)

### A HOSPITAL WEEK AND PAGEANT.

As we go to press a meeting of hospital representatives is being held (November 14) at the National Hospital, Queen Square, to receive the report of the Executive Committee which was appointed to go into the question of cost and other details. This report, if and when approved, will be circulated among the Boards of Hospitals and the Funds, and a further meeting will be held on November 28 to come to a final decision. We understand that hospital secretaries are displaying considerable keenness in the matter, and that it is very probable that a Hospital Pageant on a large scale will be one of the features of the coming year. The most recent effort, Fleet Street Week, which brought in over £5,000 for St. Bartholomew's Hospital, augurs well for the success of a grand co-ordinated campaign on behalf of all the Metropolitan hospitals, be the results expressed directly in £ s. d. or in the addition to public knowledge of the work and objects of our hospitals, to which such a campaign should primarily be directed.

## Figures and Tables

**Figure f1:**
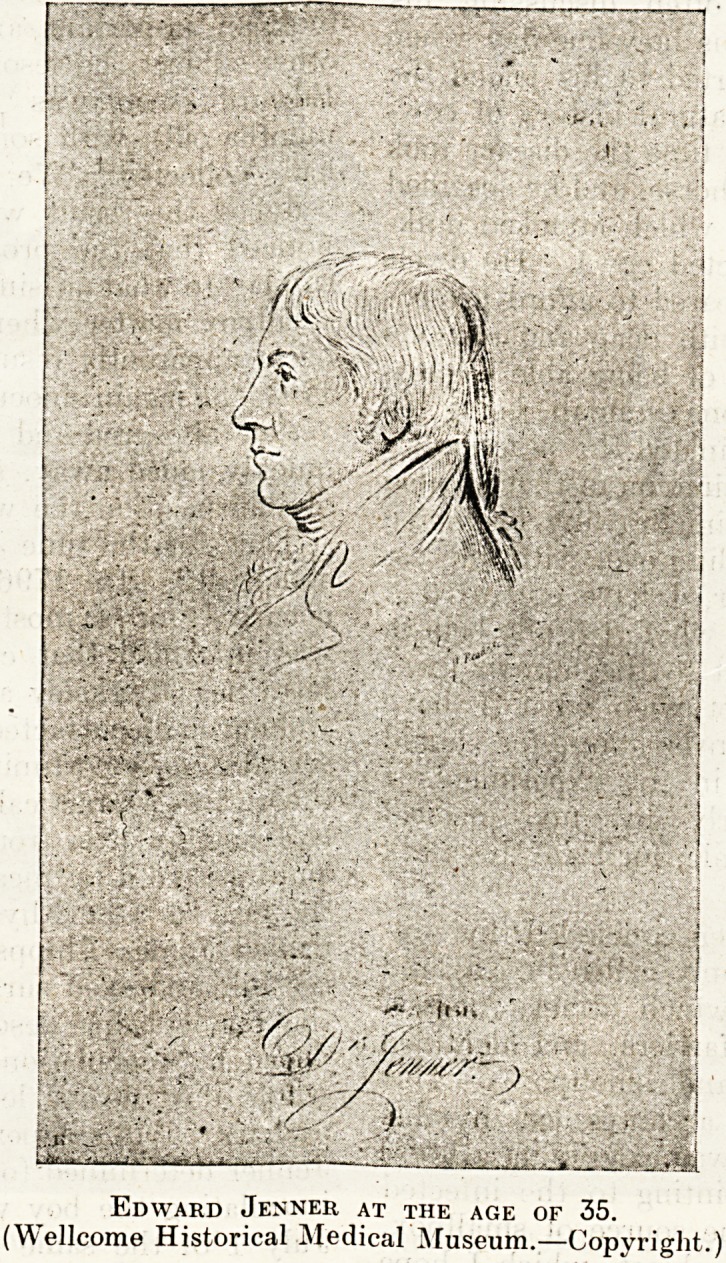


**Figure f2:**